# Systematic Review of the Relationship between Couple Dyadic Adjustment and Family Health

**DOI:** 10.3390/children8060491

**Published:** 2021-06-09

**Authors:** Nerea Jiménez-Picón, Macarena Romero-Martín, Lucia Ramirez-Baena, Juan Carlos Palomo-Lara, Juan Gómez-Salgado

**Affiliations:** 1 Centro Universitario de Enfermería Cruz Roja, University of Seville, 41009 Sevilla, Spain; nejipi@cruzroja.es (N.J.-P.); luraba@cruzroja.es (L.R.-B.); jucapa@cruzroja.es (J.C.P.-L.); 2 Faculty of Nursing, University of Huelva, 21007 Huelva, Spain; 3 Department of Sociology, Social Work and Public Health, Faculty of Labour Sciences, University of Huelva, 21007 Huelva, Spain; jgsalgad@gmail.com; 4 Safety and Health Postgraduate Programme, Universidad Espíritu Santo, Guayaquil 092301, Ecuador

**Keywords:** couple dyadic adjustment, family health, family system, systematic review

## Abstract

The importance of family functioning in the development of child and adult psychopathology has been widely studied. However, the relationship between partners’ adjustment and family health is less studied. This paper aims to describe and summarize research that analyzes the relationship between partners’ adjustment and family health. A systematic review was conducted in the PubMed, PsycINFO, Scopus, Lilacs, Psicodoc, Cinahl, and Jstor databases. Inclusion criteria were as follows: articles published from 2012 to 2019 in English, Spanish, or Portuguese. Data were extracted and organized according to the family health model: family climate, integrity, functioning, and coping. Initially, 835 references were identified, and 24 articles were assessed for quality appraisal. Finally, 20 publications were selected. Results showed that couple adjustment was an important factor that triggered the emotional climate of the family, was positively intercorrelated to parenting alliance or coparenting, and contributed to family efficacy and help when facing stressful life events. Findings revealed a consensus about the relationship between couple dyadic adjustment and family health. The results could orientate interventions to promote well-being and to increase quality of life and family strength. Health professionals should thoroughly study couple relationships to identify risk factors, assess family skills, and promote family health.

## 1. Introduction

The focus of health care was recently oriented to families [1]. This approach supposed a tactical and strategical change in the practice of health professionals. In order to achieve this new approach, it has been necessary to take into account a conceptual, theoretical, and instrumental framework, to describe the reality of families [1]. In this sense, the family is the smallest social unit, and it is considered as the main and fundamental basis of any society. It is the core of socialization, education, and optimum biopsychosocial development of its members. Family provides the context where values are transmitted, ideas are learned and adopted, and beliefs and norms of conduct are acquired [2]. Family is considered a group of two or more people coexisting as a spiritual, cultural, and socio-economic unit. Even without physical coexistence, they share psycho-emotional and material needs and common objectives and interests [3].

Traditionally, Family System Theory has been considered the theoretical framework in family research [1]. According to this theory, a family is an open system with relationship patterns at individual, dyadic, and systemic levels, with interconnectedness among the various levels. It comprises three primary subsystems (marital, parental, and sibling) that are delineated by boundaries and rules connected to themselves, which affect, and are reciprocally affected, by each other [4]. Each subsystem contributes to family functioning through an exercise of roles and tasks that are necessary for the whole system [2]. 

Family health is considered a complex concept, a multidimensional, interactive holistic phenomenon that includes the biological, psychological, spiritual, sociological, and culture factors of individual members and the whole family system [5]. Family health is the family capacity to function effectively as a biopsychosocial unit, meeting the needs of its members and contributing to their development. It is a dynamic process that requires an optimal social climate and family integrity, good family functioning, and a level of resistance and coping that allows the family to deal with crisis situations [6]. Lima-Rodríguez et al. [6,7] proposed a model of family comprised of five dimensions: climate, integrity, functioning, resilience, and coping, which are described in Table 1.

Within the family system, research has identified the couple subsystem as the principal and fundamental one for the development of family members and the maintenance of family health [8,9]. The couple dyadic adjustment has traditionally been related to health and well-being and couple dyadic maladjustment has been related to disease [10]. Greater marital quality has been related to better health, including lower risk of mortality and lower cardiovascular reactivity during marital conflict [11]. This approach has been the basis for relating the criteria of well-being or couple distress with welfare or discomfort in family health [10]. Previous studies have identified a positive link between the quality of the marital relationship and the relationship they establish with their children [12]. Parental conflict influences the children emotionally, physiologically, cognitively, and behaviorally in regard to their growth, and it has an impact on their development and mental health over time [13]. Interparental conflict also affects their parenting behaviors, particularly harsh discipline and parental acceptance [14]. So, it is essential that the couple subsystem has a good dyadic adjustment for the maintenance of family health. Recent literature suggests interventions based on the quality of the significant others’ relationship to promote family functioning are needed [15].

The quality of the relationship or the couple dyadic adjustment are indicators of the health of the couple. Couple dyadic adjustment represents the degree to which couples are satisfied with their relationship in domains such as cohesion, satisfaction, consensus, and affective expression (Table 2). Thus, high levels of dyadic adjustment reflect better adjustment and better quality of couple relationship [11,16,17,18,19,20,21].

The novelty of this study is the family approach when considering couples, including the private ambiance of the family, and considering the family health as a multiscale concept. We aim to address the knowledge gap about the relationship of couple dyadic adjustment and family health. The objective of this study is to describe and summarize research that analyzes the relationship between couple adjustment and family health.

## 2. Materials and Methods

### 2.1. Design

A systematic review was conducted according to the Preferred Reporting Items for Systematic Reviews and Meta-Analyses [22].

### 2.2. Search Methods

The following databases were consulted: Pubmed, PsycINFO, Scopus, Lilacs, Psicodoc, Cinahl, and Jstor. The search was carried out in January 2020 using the following search strategy: (conjugal or dyadic) and (“family functioning” or “dysfunctional family” or “family conflict” or “family health”).

Inclusion criteria were as follows: (a) empirical study using quantitative methodology, to obtain articles that analyze the relationship between couple dyadic adjustment and family health; (b) written in English, Spanish, or Portuguese; (c) published between 2012 and 2019 (both included), because recent scientific production leaves obsolete other previously published [23]; (d) nuclear families.

Exclusion criteria were as follows: (a) duplicated references; (b) non-access to full text article; (c) not relevant to the aim of the study, such as experiences of domestic violence or gender-based violence because of the multicausality of this issue itself; (d) studies with qualitative methodology; and (e) studies with low methodological quality after assessing the risk of bias.

Family health includes a wide range of complex concepts, such as functioning, adaptation, resilience, coping, etc. For this reason, its definition and the nature of the concept of family health is misleading and needs to be defined clearly and precisely. To make it operational, measurable, and facilitate its evaluation, it is necessary to design and use validated instruments [6]. Following the suggestion of these authors, we established as eligibility criteria quantitative studies that used measuring instruments for family health or any of its dimensions.

### 2.3. Search Outcome

The initial electronic search yielded 835 references, from which 286 were duplicates. After reading the title and abstract, 417 references were excluded, as they did not meet the inclusion criteria. Then, 132 full text articles were screened, and 92 were excluded because the full text article did not report any relationship between couple dyadic adjustment and family health. Finally, a set of 24 full text articles were potentially relevant for eligibility.

### 2.4. Quality Assessment

**Figure 1:**
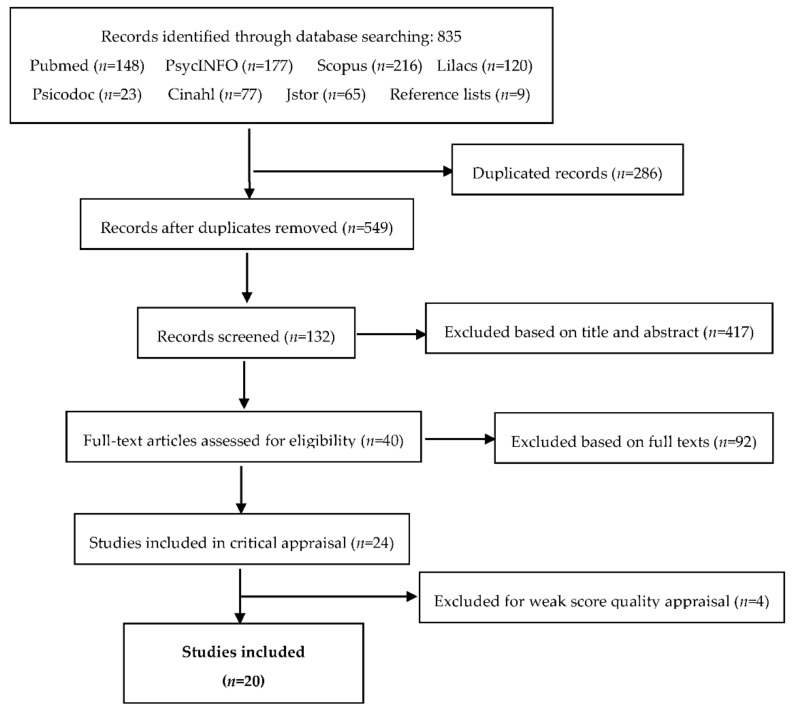
Flow diagram of the selection process.

Critical appraisal of 24 articles was conducted according to the Quality Assessment Tool for Systematic Reviews of Observational Studies Score. It assesses methodological rigor according to five items that cover for the following aspects: external validity (1 item), reporting (2 items), bias (1 item), and confounding factors (1 item). Assessed studies can be scored as poor quality; satisfactory quality; or good quality [24]. Out of the 24 articles, 4 articles scored poor, 8 scored satisfactory, and 12 scored good regarding methodological quality. Articles that scored poor in the critical appraisal were excluded. Finally, 20 articles were selected for this systematic review (Figure 1).

### 2.5. Data Extraction

A critical reading of the selected articles was conducted. Two researchers extracted key descriptive details from the studies. Data were classified regarding authors, year of publication, country, aim, theoretical background, methodology (type of study, participants, data collection, and data analysis) and main findings. Ad hoc forms were created where researchers independently and by pairs included statistical results of each study. These forms were compared in order to resolve discrepancies between reviewers. When discrepancies were found, they were subject to review within the research group until consensus was reached.

### 2.6. Synthesis

It was agreed to adopt a data-driven thematic analysis. Results confirming a relationship between couple dyadic adjustment and family health were presented according to five categories identified by Lima-Rodríguez et al. [6,7]: climate, integrity, functioning, resilience, and coping. The review process, data extraction, and analysis were carried out by two independent researchers.

## 3. Results

### 3.1. Study Characteristics

The characteristics and main results of the reviewed studies are summarized in Table 3 [25,26,27,28,29,30,31,32,33,34,35,36,37,38,39,40,41,42,43,44]. Most of the studies followed a family approach within a theoretical framework; the Family System Theory was the most used. Two of the studies combined several theories to describe family approach [29,34], and three of them did not state the theoretical model used [25,31,41]. The majority of studies were conducted in the United States. Most of the studies were cross-sectional and prospective versus longitudinal or cohort study. The 20 studies addressed the couple subsystem from the perspective of dyadic adjustment (*n* = 10), the satisfaction (*n* = 5), or the conflict (*n* = 5). There was no uniformity in the term employed. Only six of the studies used the Dyadic Adjustment Scale (DAS) (Table 1).

### 3.2. Relationship between Couple Dyadic Adjustment and Family Climate

Results showed that the quality and satisfaction of the couple were important factors that can act as a trigger for the emotional climate of the family and affected the home learning environment [29]. The couple satisfaction was associated with positive family emotional expressiveness [29]. The quality of the couple relationship showed an indirect effect on parent–child relationship quality mediated by coparenting perceptions [44]. In this sense, greater couple dyadic adjustment was related to less family conflict, so when there was low couple dyadic adjustment, it was related to family aggression (verbal and physical acts of aggression) [40]. More so, partners’ conflict was related to higher levels of children’s temperamental emotionality [28,40], i.e., children as being more overtly and more relationally aggressive [28]. Dyadic adjustment predicted family conflict in children at age 3 years [28] and 5 years [40]. Dyadic adjustment also predicted lower aggressive behavior among adolescents [42].

### 3.3. Relationship between Couple Dyadic Adjustment and Family Integrity

Family integrity is considered to be the support and engagement behavior established among family members. Results demonstrated that couple adjustment was positively related to parenting alliance [26] or coparenting [26,37,41]. In this sense, parenting alliance was positively related to all dimensions of dyadic adjustment [26]. Partners’ satisfaction and couple relationship maintenance were positively related to supportive coparenting and negatively related to undermining coparenting in the couples with children aged from 9 to 13 years [36,37]. Couples with conflicting relationships based on negativity showed less support and more hostility in coparenting [41].

### 3.4. Relationship between Couple Dyadic Adjustment and Family Functioning

The couple dyadic adjustment was related to family functioning. On one hand, dyadic security, dyadic conflict, and couple relationship quality were related to interaction and family structure [32]. On the other hand, high conjugal adjustment was related to family flexibility and family satisfaction, and low couple dyadic adjustment was related to family disengagement and chaos [25]. It is necessary that family members contribute to proper functioning of the family unit. In this sense, the results revealed the relationship between couple dyadic adjustment and parental role performance [26,28,36] and in child functioning [25,30,31,34,35,38,41]. So, when couples engaged in activities to maintain their relationship and in actions to improve couple satisfaction, the effectiveness of their parental practices was greater than in those who did not invest in their relationship [36]. The couple conflict appeared positively related to parenting stress [26] and to more punitive and hostile parenting behaviors [28]. Similarly, lower couple dyadic adjustment was related to higher levels of children’s temperamental behavior [30], with problems in internalizing symptoms [43], internalizing and externalizing sadness, anger, and emotional reactions [31,34], hyperactivity and difficulties [25], difficult behavior with fathers [38], child negativity behavior [41], and suffering from psychopathological symptomatology i.e., anxiety, depression, withdrawal, somatic, attention, aggression, and delinquency in adolescents and young adults [31,35].

### 3.5. Relationship between Couple Dyadic Adjustment and Family Coping

When facing stressful life events, a family must rely on its ability and resources to address them. The reviewed articles studied couple dyadic adjustment during the prenatal phase [30], the birth of the first child [33,39], and transition to parenthood [27]. The results described that when couples exhibited a higher quality of couple interaction prior to the birth of their infant, they also showed more supportive coparenting behavior postpartum [39]. When couples exhibited higher conjugal satisfaction, they also showed more positive parenting experience, more parenting sense of competence and more positive parenting life change [33]. On the other hand, during the prenatal phase, couples that exhibited tension due to prolonged silences, stiff postures, and lack of eye contact, whining, or personal attacks, they showed higher coparenting conflict, pervasive disagreements, and higher use of hostility, sarcasm, and insulting behavior among family members [30]. In the prenatal stage, couples with a negative affective relationship were related to the emotional withdrawal in children at the age of 8 months and coparenting conflict at children aged 24 months [30]. In the transition to paternity, couple conflict predicted lower satisfaction, competitive and cooperative coparenting, and higher involvement in parenting [27].

## 4. Discussion

This systematic review identified the relationship between couple dyadic adjustment and family health within the domains of climate, integrity, functioning, and family coping. No results were identified related to family resilience. Results showed that greater couple dyadic adjustment was related to the higher emotional climate of the family, i.e., less family conflict and family aggression, higher levels of home learning environment, and positive family emotional expressiveness. These results are consistent with previous studies. Ackerman et al. [8] found that 37.3% of the variables explained by family climate are due to dyad adjustment. Schermerhorn et al. [9] identified a positive relationship between Dyadic Adjustment Scale (DAS) and Family Environment Scale. Other authors attribute couple quality to the family emotional environment [45]. A recent review of the literature regarding couple relationships and children’s adjustment revealed that children growing up in a conflicting environment are at risk for problematic development [46].

Regarding family integrity, results showed that couple satisfaction and couple relationship maintenance were related to parenting alliance and supportive coparenting. Coparenting has been defined as a shared involvement from parents in children’s education, rearing, and life decisions [47]. Couple satisfaction affects coparenting quality in terms of involvement and cooperative coparenting, and therefore, children’s development [48]. Results are in line with the reflections of Boehs et al. [49] on the routines and rituals of the family, which is part of family integrity. They stated that they are constantly modified to meet basic needs of family members such as a spouse´s needs or the upbringing of children. Other authors related dyad disadjustments to couple dissolution or similarly, the end of family integrity through divorce or separation [50].

Higher couple dyadic adjustment was related to family efficacy, family flexibility, family satisfaction, and good performance of family member’s roles for proper functioning of the family unit. Low couple dyadic adjustment was related to family disengagement, chaos, parenting stress, and more punitive and hostile parenting behaviors. The authors have previously described a relationship between couple adjustment and family functioning [51]. Eichelsheim et al. [52] assumed that couple dyad members had to make adjustments not to alter relationships with other members of the family and the family functioning.

The reviewed studies did not address the relationship between couple dyadic adjustment and family resilience. Gómez and Kotliarenco [53] identified those environments that activate family resilience to recuperate optimum functioning level and common welfare: situations of chronic risk, significant crisis, or family tension.

Finally, the couple dyadic adjustment was related to family coping when there were problems or stressful life events. In this case, the reviewed articles studied couple dyadic adjustment during the prenatal phase, the birth of the first child, and the transition to parenthood. When couples exhibited higher satisfaction with their relationship, they also showed more positive parenting experience, more parenting sense of competence, and more positive parenting life change. Previous studies related higher couple quality with less conflicts and with more positive results in resolving family coping [54]. In this sense, recognizing difficult situations for couples to deal with would be the first step in coping and looking for advice provided by via education programs or through personal, family, or couples therapy [55].

For more than three decades, researchers such as Driver et al. [56] have studied the interactional patterns of couples’ success or failure. The success of a partners’ relationship depends primarily on how the couple handles conflicts. The way a conflict is used and resolved in the couple dyad relationship suggests the health and longevity of the family unit because healthy couples try to repair their relationship [57]. For many researchers, dyadic adjustment appears to be an important aspect of well-being [58]. To many researchers, the couple relationship is the basis of the family, its relational center [59,60], and the key organizational factor of family life [50]. Olson and Gorall [61] merged the concepts of couple and family dynamics in the Circumplex Model of Marital and Family Systems. They considered that balanced families will function more adequately across the family life cycle and tend to be healthier families. They considered the couple subsystem as the leader within the family with the ability to demonstrate flexibility in roles, relationships, and rules, including control, discipline, and role sharing to adapt to stressors, thus promoting family health. Other researchers also identified the partners’ relationship as the principal variable and fundamental for the maintenance of family health [8,9].

Family health research is of great interest to many health professionals because family plays an essential role in the health and illnesses of people [62], and because the family should be considered as a system in which the interactions between family members have to be the target for the health interventions. However, there is a lack of care and attention from health professionals when dealing with family units [63]. They highlight the lack of a theoretical model or a strategic diagnosis and evaluation plan nor care or efficient treatment in the environment of family health. Researchers agree to define family health as a changing and dynamic status that allows growth and satisfaction to family members’ ‘needs and family itself’. It also allows the interaction between individual, family and society, problem solving, skills to manage changes and/or stressful events as well as to adapt to crisis situations [5]. In spite of all these, few clinical research studies employ the family health variable as a study target as well as a multiscale concept. The Lima-Rodriguez model [6,7] based on five interconnected dimensions could be a valid proposal for future studies investigating family health.

Health professionals would derive value from being qualified and trained to interact with the family and aim to optimize its health. Their responsibilities could include a thorough assessment of the family as a unit [64], identifying family problems as well as the availability of resources, and delivering customized education for families to identify their strengths and overcome new threatening situations or continue meeting their health needs [15].

### Limitations

The present review may contain biases. Inclusion criteria such as language, the period of time, or quantitative design may have conditioned the identification and recovery of relevant articles. However, in order to minimize bias, the references of the selected studies were checked to include other results to our review, and we addressed the results and their categorization through consensus and individual and group analysis. Another limitation that should be acknowledged is that this review focuses on couple dyadic adjustment as opposed to other forms of caregiving relationships. There are a wide variety of families and situations that are not reflected in this systematic review that perhaps could give a wider approach and enriches the results from this review. Likewise, we did not take into consideration the families with children with special needs/disabilities, as results only refer to typically developed children. By establishing quantitative methodology studies as the conclusion criterion for this review, the results obtained from a qualitative approach were excluded. Due to the diversity of current families and the strong cultural component of the concept of family health, a qualitative approach is essential for a complete understanding of the phenomenon. So, qualitative research in this area would also be useful in combination with quantitative work.

## 5. Conclusions

Findings from this review revealed a consensus about the relationship between couple dyadic adjustment and family health. Although the leading role of the couple within the family has been extensively studied, this review fills the gap of knowledge about the relationship between dyadic adjustment and family health. The originality of this study also lies in the multidimensional approach to the concept of family health compared to previous reviews.

These results could help to develop strategies to improve the quality of the couple relationship and to contribute to a good family climate. As couple dyadic adjustment can aggravate family integrity, health professionals should care for family susceptibility and vulnerability in this sense. In addition, they could focus their interventions on the couple subsystem to improve the specific areas of family functioning: affective involvement, roles, communication, and problem solving. On one hand, the couple subsystem has knowledge, experiences, and competences that can contribute to family resilience. On the other hand, when the couple enters into conflicts, the family may lose some of these resources and become fragile in order to face of the impact of stressors. Finally, the challenges of the couple relationship could trigger processes of family coping. Caring for the couple subsystem could promote family health and foster the development of its members.

## Figures and Tables

**Table 1:** Dimensions of family health.

Dimension	Description
Family climate	The internal family environment. It depends on the communication and relationships among its members, the type of cohesion, and the stability of the system.
Family integrity	The degree of union between family members. It depends on the level of commitment, involvement, and family loyalty. The involvement to solve problems and share concerns and feelings.
Family functioning	It is oriented to needs satisfaction, family processes development, and adaptation to changes. Family functioning depends on the composition and structure, family organization, role performance, the adequacy of rules, the pattern of communication and relationships, and the maintenance of a clear line of authority.
Family resilience	It contributes to strengthening of the family and depends on knowledge, experiences, and internal and external resources available.
Family coping	It is related to the family’s ability to deal with a problem or stressful life events.

**Table 2:** Dimensions of couple dyadic adjustment.

Dimension	Description
Cohesion	The degree of agreement between partners regarding shared activities.
Satisfaction	The low incident rate of quarrels, discussions of separation, and negative interactions.
Consensus	The degree of agreement between partners regarding different aspects of their lives such as those involving money, friends, household tasks, and time spent together.
Affective expression	The satisfaction level regarding sexuality and manifestations of tenderness.

**Table 3:** Characteristics and main results from the reviewed studies.

Authors (Year) Country	Aim and Theoretical Background	Study Design and Methods: Type of Study, Participants, Data Collection	Results of Studies Organized into Thematic Categories
Baiocco et al. (2015) Italy [25]	To compare homosexual and heterosexual parent families on dyadic and family functioning. Unidentified.	Cross-sectional and prospective: 40 homosexual couples and 40 heterosexual couples. Questionnaires: DAS, Family Adaptability and Cohesion Evaluation Scale, Emotion Regulation Checklist, and The Strengths and Difficulties Questionnaire.	Family Functioning. Dyadic adjustment was associated with family flexibility, family disengaged, chaotic family, and family satisfaction. Dyadic adjustment was associated with children hyperactivity and difficulties.
Camisasca et al. (2014) Italy [26]	To explore the parenting alliance on the relationship between marital adjustment and maternal and paternal stress. Family System Theory.	Cross-sectional and prospective: 236 families with children aged from 6 to 11 years. Questionnaires: DAS, Parenting Alliance Measure and Parenting Stress Index Short Form.	Family Integrity. Parenting alliance was correlated to dyadic adjustment of both wife and husband (consensus, satisfaction, affectional expression, and cohesion) Dyadic adjustment predicts parenting alliance in wife. Family Functioning. Parenting stress was correlated to dyadic adjustment of both wife and husband (consensus, satisfaction, affectional expression, and cohesion).
Christopher et al. (2015) USA [27]	To examine changes in first-time parents’ marital quality over the transition to parenthood as predictors of their coparenting quality. Family System Theory.	Longitudinal: 125 couples in the transition to paternity. Videotaping and Questionnaires: Relationship Questionnaire, Coparenting and Family Rating scales, and Marital Opinion Questionnaire.	Family coping. In the transition to paternity, the following correlations were found: satisfaction and competitive coparenting; involvement in parenting and cooperative coparenting and husband’s support of partner´s parenting. Marital conflict predicted lower cooperative coparenting.
Doh et al. 2012) Korea [28]	To examine the relationship between marital conflict, child maltreatment, and young children’s aggressive behavior. Family Ecology Model.	Cross-sectional and prospective: 349 mothers with 3-year-old children. Questionnaires: Children’s Perception of Interparental Conflict, Parent-to-Child version of the Conflict Tactics Scale, and Preschool Social Behavior Scale—Parent Form.	Family Climate. Marital conflict was correlated to children being more overt and relationally aggressive. Family Functioning. Those who reported more frequent and severe marital conflict were more likely to neglect their children.
Froyen et al. (2013) USA [29]	To investigate associations among marital satisfaction, family emotional expressiveness, the home learning environment, and emergent literacy. Family Systems and Human Ecological Theories.	Cross-sectional and prospective: 385 two-parent families and son/daughter adolescent. Questionnaires: Kansas Marital Satisfaction Scale, Family Expressiveness Questionnaire—Short Form and Parenting Questionnaire.	Family Climate. The marital satisfaction significantly predicted both positive family emotional expressiveness and negative family emotional expressiveness. Marital satisfaction was related to emotional expressiveness in the home and with the home learning environment and children’s literacy skills. The model provided an adequate fit to the data.
Gallegos et al. (2016) USA [30]	To investigate whether prenatal marital negative affect spills over to parents’ emotional withdrawal in interactions with their infants. Family System Theory.	Observational and longitudinal: 125 couples that were expecting their first child. Videotaping.	Family Functioning. Marital negative affect was associated with infant temperament. Family coping. Prenatal marital negative affect was associated with both wife and husband toddler emotional withdrawal at child age 8 months and coparenting conflict at child age 24 months.
Hayatbakhsh et al. (2013) USA [31]	To examine whether family structure and the quality of the marital relationship have a long-term impact on offspring’s psychopathology in early adulthood. Unidentified.	Cohort, prospective. 3473 young adults with parents with first marriage. Questionnaires: DAS and Young Adult Self-Report.	Family Functioning. Marital conflict was associated with deterioration of offspring’s behavior (anxiety/depression; withdrawal; somatic; attention; aggression; delinquency; internalizing; and externalizing)
Jager et al. (2014) USA and Europe [32]	To examine whether each dyad member’s unique perspective of family dysfunction is associated with the shared dyad perspective of dyad adjustment. Family System Theory.	Cross-sectional and prospective. 128 two-parent families with adolescent members. Self-Report Measures of Family Dysfunction and DAS.	Family Functioning. Family and unique perspectives of family dysfunction predicted dyadic perspectives of dyadic adjustment. Family perspective of family interaction and family structure were related to dyadic security; to dyadic conflict; and to marital quality.
Kershaw et al. (2014) USA [33]	To assess the influence of relationship and family factors during pregnancy on parenting behavior 6 months postpartum. Ecosystem Model.	Longitudinal. 296 pregnant adolescents and their male partners. Interviews via audio computer-assisted self-interview.	Family coping. Higher couple relationship satisfaction during pregnancy was related to more parental involvement at 6 months postpartum; more positive parenting life change; more positive parenting experience; and more parenting sense of competence.
Lindahl et al. (2012) USA [34]	To teste whether disturbances in family subsystem alliances would be related to child maladjustment. Structural and Family System Theory.	Cross-sectional, prospective. 270 couples with children aged from 6–12 years. Videotaping and Questionnaires: Child Behavior Checklist and System for Coding Interactions and Family Functioning.	Family Functioning. If there is imbalance in the couple subsystem, the offspring have problems in internalizing behavior via sad affect and via angry affect and externalizing behavior via angry affect.
Melo et al. (2014) Portugal [35]	To analyze to what extent interparental conflicts act as predictors of psychopathological development in young people. Ainsworth’s attachment theory.	Cross-sectional and prospective. 827 participants with children. Questionnaires: Sociodemographic Questionnaire, Children’s Perception of Interparental Conflict Scale and Brief Symptom Inventory.	Family Functioning. The frequency and intensity of marital conflicts predicting variables of depression and anxiety in adolescents and young adults. The model explains 10.2% of the total variance.
Merrifield et al. (2013) USA [36]	To examine the associations among marital quality, coparenting, and parenting self-efficacy in parents of young children. Family System Theory.	Cross-sectional and prospective. 175 couples with children. Questionnaires: Maintenance Scale, Kansas Marital Satisfaction Scale, Family Experiences Questionnaire and Parenting Self-Efficacy Scale.	Family Integrity. The satisfaction marital was correlated to supportive coparenting of both husband and wife and undermining coparenting. The conflict marital was correlated to supportive coparenting and undermining coparenting. The marital relationship maintenance was correlated to supportive coparenting and undermining coparenting. Family Functioning. Parenting self-efficacy was correlated to marital satisfaction, marital conflict, and marital relationship maintenance. The regression model was formed with control variables, marital qualities, and supportive coparenting, and it was significantly associated with parenting self-efficacy in wife and husband.
Pedro et al. (2012) Portugal [37]	To explore if marital satisfaction and contributions to coparenting may be important to support and maintain partners’ positive parenting practices. Family System Theory.	Cross-sectional and prospective. 519 couples with children aged from 9–13 years. Questionnaires: Marital Life Areas Satisfaction Evaluation Scale, Coparenting Questionnaire and Portuguese version of the EMBU (Egna Minnen Beträffande Uppfostran).	Family Integrity. The marital satisfaction was correlated to the coparenting and coparenting conflict. The fitted model indicated that both parents’ contributions to cooperation and conflict were intervening variables in the relationship between his or her marital satisfaction and the partner’s parental practices, and paternal parenting model. Marital satisfaction explained between 35% and 38% of the variance of cooperation.
Shigeto et al. (2014) USA [38]	To investigate if child’s difficult temperament moderates the link between family cohesiveness/marital adjustment and child behavior. Family System Theory.	Longitudinal. 59 pairs of mother-child and father–child dyads. Videotaping. Correlation and regression	Family Functioning. Marital adjustment and children’s difficult behavior with fathers were both significant.
Shoppe-Sullivan et al. (2013) USA [39]	To examine parent characteristics as correlates of coparenting behavior in primiparous couples. Family System Theory.	Longitudinal. 57 primiparous couples. Videotaping and Questionnaires: Multidimensional Personality Questionnaire, ‘What is a father’ questionnaire and Mother–Father—peer scale.	Family coping. Marital behavior was associated with supportive coparenting behavior. When couples exhibited higher quality marital interaction prior to the birth of their infant, they also showed more supportive coparenting behavior postpartum.
Soo-Yoo et al. (2015) USA [40]	To examine the impact that fathers’ experience of distress has on the overall emotional climate within their families. Belsky’s model of parenting and attachment theory.	Longitudinal. 319 fathers and their twins. Questionnaires; DAS, Eysenck Personality Inventory, Family Environment Scales, Colorado Childhood Temperament Inventory and MacArthur Story Stem Battery.	Family Climate. Dyadic adjustment was correlated to family aggression, family conflict, negative disciplinary representations (verbal and physical acts of aggression such as demeaning, hitting, pushing, kicking, or killing), and children’s temperamental emotionality, and it predicts family conflict at child age 5 years.
Stapleton et al. (2012) USA [41]	To examine if marital satisfaction influences of children’s behavior. Unidentified.	Longitudinal. 84 couples. Videotaping and Questionnaires: Demographic Questionnaire, Kategoriensystem fuer Partnerschaftliche Interaktion, Marital Adjustment Test and Parent–Child Structured Interaction Qualitative Rating Scales. Correlations, regression and actor–partner interdependence modeling	Family Integrity. Marital conflict positivity and support positivity was correlated to parent support and parent hostility. Marital conflict negativity and support negativity was correlated to parent support and parent hostility. Family Functioning. Marital support positivity was correlated to child negativity behavior. Marital support negativity in marital relationship was correlated to child negativity.
Smokowski et al. (2017) USA [42]	To examine the influence of family functioning, including parent–adolescent conflict, parent worry, and parent marital adjustment, on aggression among Latino adolescents. Family Coercion Theory of Childhood Aggression.	Longitudinal. 258 pairs adolescent-parent. Child Behavior Checklist, Youth Self-Report, Conflict Behavior Questionnaire-20, Penn State Worry Questionnaire, Dyadic Adjustment Scale.	Family Climate. Dyadic adjustment was a significant factor associated with decreased aggressive behavior.
Mosmann et al. (2017) Brazil [43]	To evaluate the associations of marital, parenting, and coparenting with internalizing and externalizing symptoms in children. Feinberg model of coparenting.	Descriptive, cross-sectional, quantitative. 200 participants. Family Adaptability and Cohesion Evaluation Scale, Marital Conflict Scale, Parental Practices Scale, Coparenting Relationship Scale, Child Behavior Checklist.	Family Functioning. Two variables were revealed as predictors of internalizing symptoms: marital adaptability and coparental approval, providing an explained variance coefficient (R^2^) of 0.134, determining that the predictor variables explain 13.4% of internalizing symptoms.
Holland et al. (2013) USA [44]	To examine coparenting perceptions of support and trust as a link between marital quality and parent-child relationship. Family systems theory.	Descriptive, cross-sectional. 122 families. Strange situation procedure, Child-Parent Relationship Scale, Intimate Relationships Scale, Parenting Alliance Inventory. Multiple correlation.	Family Climate. Coparenting revealed as a link between marital quality and parent–child relationship quality. The fitted model indicated an indirect effect of marital quality on parent–child relationship quality via coparenting perceptions.

## Data Availability

Not applicable.
